# Surgical Decision Making in Preduodenal Portal Vein: Report of Two Cases in Neonates

**DOI:** 10.1055/s-0038-1661409

**Published:** 2018-07-05

**Authors:** Simona Rusu, Ahmad Zaghal, Muhammad S. Choudhry

**Affiliations:** 1 Department of Pediatric Surgery, Chelsea and Westminster Hospital NHS Foundation Trust, London, United Kingdom of Great Britain and Northern Ireland

**Keywords:** Preduodenal Portal Vein, atrial isomerism, duodenal obstruction, malrotation, duodenoduodenostomy

## Abstract

Preduodenal portal vein (PDPV) is a rare anomaly that can cause duodenal obstruction. PDPV is associated with other congenital anomalies, mainly cardiac and gastrointestinal. Treatment usually consists of bypassing the obstruction by duodenoduodenostomy. We report two cases of PDPV in association with atrial isomerism and malrotation with different surgical management.

## Introduction

Preduodenal portal vein (PDPV) is a rare anomaly in which the portal vein is located anterior to the second part of the duodenum and represents 4% of all cases of duodenal obstruction. 1 This anatomical abnormality can result in an upper gastrointestinal tract partial or complete obstruction. This anomaly was first described by Knight in 1921. 2 PDPV is associated with a wide variety of congenital malformations such as dextrocardia, atrial isomerism, malrotation, situs inversus, duodenal atresia, biliary atresia, pancreatic anomalies, and polysplenia. 3 4 5 6 PDPV may have developed due of abnormal involution of the vitelline veins or due to an abnormal rotation of the gastroduodenal loop as it is a frequent association with malrotation, or a combination of both. 2 7 8

Here, we report two cases of intestinal malrotation in conjunction with cardiac abnormalities and an incidental finding of PDPV. Different surgical strategies were applied to manage these two patients, with good outcomes.

## Case 1

A 14-day-old baby girl was referred to our surgical team due to ongoing nasogastric bilious aspirates. Patient was born at 31 weeks of gestation in a poor general condition, initially required intubation with high frequency oscillatory ventilation, inotropic support, and surfactant administration. She had an antenatal diagnosis of left atrial isomerism, dextrocardia, and a right-sided stomach query situs inversus, which were confirmed postnatal. Enteral feeds were started on day 2 of life with maternal expressed breast milk via a nasogastric tube (NG) but had difficulties in reaching full feeds. She passed meconium on day 3 of life.

**Fig. 1:**
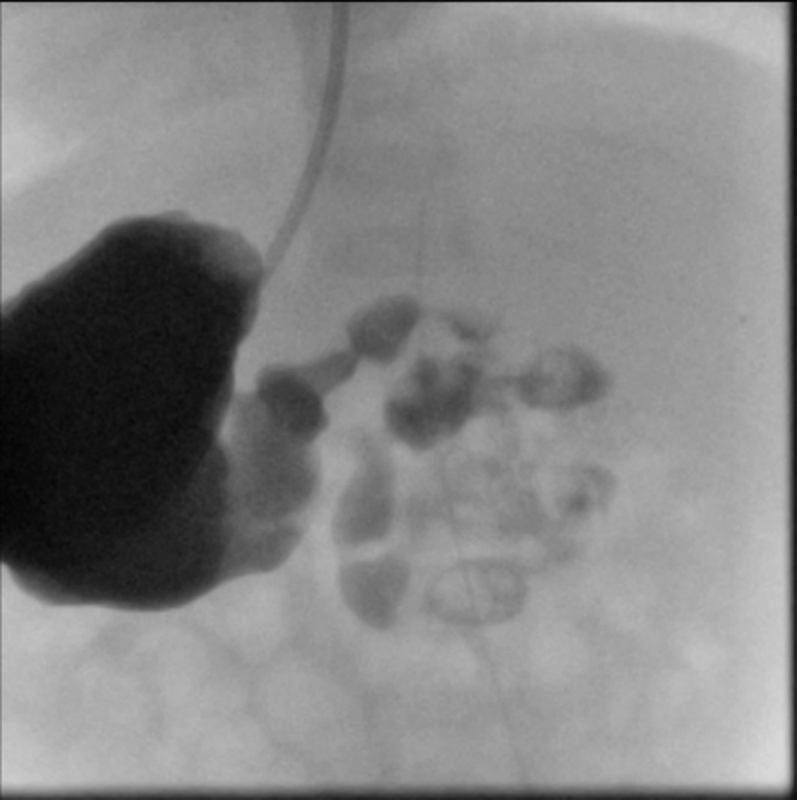
Upper gastrointestinal contrast study showing a right-sided stomach and an abnormal position of the duodenojejunal flexure, suggestive of malrotation.

On physical examination, no gross phenotypic anomalies were noted, abdominal exam was unremarkable. Plain abdominal X-ray showed a normal gas pattern in consistent with situs inversus. An upper gastrointestinal contrast study was suggestive of intestinal malrotation: duodenojejunal flexure was demonstrated to the left of the midline with the stomach on the right; the proximal small bowel was on the left side of the abdomen ( Fig. 1 ).

**Fig. 2:**
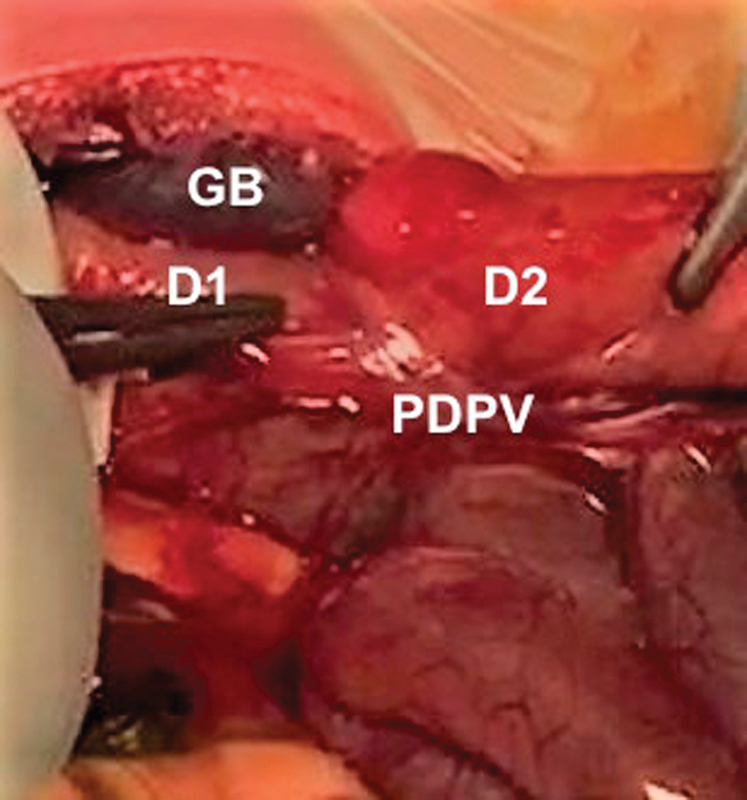
Intraoperative aspect: preduodenal portal vein crossing the second part of the duodenum. D1, first part of the duodenum; D2, second part of the duodenum; GB, gallbladder; PDPV, preduodenal portal vein.

Baby underwent an emergent laparotomy via a left upper quadrant incision. Exploration of the abdomen revealed a right-sided stomach, adhesion bands between cecum and duodenum, a broad base mesentery, and a PDPV crossing anteriorly at the level of second part of the duodenum. Ladd's bands were divided and the patency of the duodenum was checked by injecting 50 mL of air via the NG. The stomach and duodenum proximal to the PDPV were distended adequately by air; however, the duodenum distal to the aberrant crossing vein remained collapsed signifying the presence of extrinsic compression ( Fig. 2 ). A decision of duodenoduodenostomy was made and a diamond-shaped anastomosis performed anterior to this aberrant vein using 6/0 polydioxanone interrupted sutures. Air was injected again which passed distally without any hold up, showing resolution of the obstruction.

Patient had an uneventful postoperative course. Enteral feeds were commenced via NG on the second postoperative day and gradually build up to full feeds in 2 weeks. She was feeding well, opening bowels regularly, without any episodes of vomiting, and gaining weight adequately at 3 months and 1 year of age on clinic review.

## Case 2

A 1-month-old baby girl was referred to our surgical team due to recurrent nonbilious vomiting and inability to reach full enteral feeds. Patient was born at 38 weeks of gestation via an elective cesarean section with an antenatal diagnosis of congenital heart block and complex cardiac structural anomalies (left atrial isomerism, atrioventricular septal defect, dysplastic pulmonary valve, pulmonary stenosis, large patent ductus arteriosus, hypertrabeculated left ventricular myocardium). She was hemodynamically stable at birth, and commenced on enteral feeds on day 1 of life. She had an episode of suspected necrotizing enterocolitis on day 3 of life, and was kept nil by mouth, and received a 7-day course of intravenous antibiotics. Enteral feeds were restarted, but she was unable to reach full feeds. A cardiac pacemaker was inserted in the second week of life due to congenital heart block.

An upper gastrointestinal contrast study was performed, which was difficult to interpret in the presence of a large pacemaker in the epigastric region; however, the aberrant position of the duodenojejunal flexure and small bowel on the right side raised suspicion of malrotation.

Exploratory laparotomy was performed at the age of 2 months, being unfit due to cardiac status earlier. Abdominal exploration revealed malrotation, with a narrow mesentery and PDPV. Ladd's bands were divided and 50 mL of air was injected via NG tube. There was no evidence of obstruction or hold up at the level of aberrant crossing vein. Duodenoduodenostomy was not performed due to lack of any evidence of duodenal obstruction at this level.

Postoperative course was complicated by Staphylococcus epidermidis line sepsis/suspected necrotizing enterocolitis and was treated with 10-day course of intravenous antibiotics. Feeds were recommenced once recovered from this illness and gradually increased. Patient was discharged home on full enteral feeds on postoperative day 21. She was tolerating full feeds, adequately gaining weight, with no clinical symptoms of intestinal obstruction at 17 months' follow-up. She is still awaiting further cardiac surgery.

## Discussion

PDPV is a rare cause of congenital duodenal obstruction, far less common than duodenal atresia or malrotation. The number of cases reported in the medical literature among all ages do not exceed 100 cases, of which less than 50% can be labeled as obvious causes of obstruction. 6 9 10 Diagnosis is usually delayed until the time of the surgical exploration for duodenal obstruction. Esscher et al, in his review of 54 cases of PDPV, found 30% suggestive of obstruction, but only 5% were confirmed as a cause of extrinsic obstruction. 10 The association of a major cardiac anomaly with duodenal obstruction should raise the suspicion for a possible PDPV. 6 7 10 The diagnosis of PDPV is usually done intraoperative. This can be explained by the rarity of this type of duodenal obstruction and lack of specific clinical and radiological signs. Only one case has been reported in the literature where an antenatal diagnosis of PDPV was suspected and confirmed postnatally. 11

Both cases in this report had similar clinical presentation with early onset of vomiting and feed intolerance. In both cases, the PDPV was associated with cardiac abnormalities and abnormal rotation of the gastrointestinal tract. Neither of babies had an antenatal diagnosis of duodenal obstruction; no polyhydramnios and no double-bubble was noticed.

The proposed treatment of PDPV in published literature is to bypass the obstruction by performing duodenoduodenostomy. Both of our cases had malrotation and underwent Ladd's procedure; however, different surgical strategies were considered in relation to the management of PDPV. In the first case, the injection of air via the NG tube revealed significant obstruction caused by the PDPV, with air distending the gastric antrum and first part of the duodenum and failure to pass beyond. A duodenoduodenostomy was performed in this case. In the second case, however, the injection of air via the NG tube failed to show any evidence of duodenal obstruction. No duodenoduodenostomy was performed in this situation as it was not indicated. Although, in both of our patients a PDPV was identified at operation, the intraoperative assessment confirmed duodenal obstruction in only one case. The fact that both patients are currently tolerating feeds and thriving, suggest that individual assessment should be performed in case of PDPV finding and not to proceed with the bypass in all.

## Conclusion

We suggest looking for PDPV intraoperatively in cases of duodenal obstruction with associated cardiac anomalies, especially atrial isomerism. Intraoperative assessment is very important before proceeding for corrective anastomosis. Duodenoduodenostomy is the preferred procedure if extrinsic compression is confirmed.

## Notes

New Insights and the Importance for a Pediatric Surgeon Preduodenal portal vein is a rare congenital anomaly, but it needs to be considered and recognized intraoperatively, especially when duodenal stenosis or obstruction is associated with cardiac malformation. Individual appropriate surgical management should be taken and consider duodenoduodenostomy only in cases of proven cause of obstruction.
